# Communication

**Published:** 1902-01

**Authors:** 


### COMMUNICATION.

DECEMBER 5, 1901.

DEAR SIR I made up my Mind to go to learn Somting and so I mend I was going to go to school and learn to be Horse Doctor Traid. Im 19 years old and I can Read ore write ore Spell. Not Extra good But you Justt about Think how young Lads Can Read If they never Care to Learn anything. But I can Learn good. If I want to. and so I want to know How them things stand. How Much would that Cost Me the Week board and all. and How Long would It Take me to Learn the Trade. You see Ill have to know a little about it. so when I come up so that I know where I have to go. If got a good Mind to go Today and so I would like to know How it is with the school when it starts and when it quits or goes out is what I mean so Pleas let me know How it is with everything Ill go Just as quick as the school starts. So that will be about What I know for this time. so Let me know How high it comes for a man in a Week Board and all. and How long it takes to be a good horse doctor. But dont Pay any atantion on that asking Ill come of course Ill come Just as quick as you want me or you can use me. I found out of the Colage in ——. I was after that business always. But I could never Catch on to it. and Now Ill stick on to it dont you forget it. I am young yet and I am Just in my write and my best years to Learn and I will learn Like the number 66. So Please advice Me everything What I asked you Please Mr. Doctor —— of —— Told me about this school in the City of ——.
and Doctor —— said so Id get a book and then I could look Things over a little and Learn Somting about it So Please Advise me every thing about what I asked you Please
Yours very Truly
—— ——
of —— —— ——
address
so if I would get one of them books that is Where the Horse is Drawed on of Course I cant learn anything. but Just to get the Name of the whole Horse. the vains and little of everything. So be so Kind and sent me one and write me soon. So That will be all and write me soon. if there is no school now or this Winter. then I will go to —— and work in the Blacksmith shop so Id like to know as quick. as I can. then I can earn a little Money yet.

Yours Truly

